# High resolution spatial analyses of trace elements in coccoliths reveal new insights into element incorporation in coccolithophore calcite

**DOI:** 10.1038/s41598-020-66503-x

**Published:** 2020-06-17

**Authors:** Cinzia Bottini, Monica Dapiaggi, Elisabetta Erba, Giulia Faucher, Nicola Rotiroti

**Affiliations:** Università degli Studi di Milano, Dipartimento di Scienze della Terra, Milano, 20133 Italy

**Keywords:** Pollution remediation, Environmental impact, Environmental chemistry, Marine chemistry

## Abstract

Coccolithophores are phytoplanktonic algae which produce an exoskeleton made of single platelets of calcite named coccoliths. They are widespread in all oceans and directly impact the short- and long-term C cycle. The study of coccolith size, morphology and elemental composition reveals important information regarding the ability of the cell to calcify and on the factors that influence this process. In this regard, very little is known about coccolith composition and its changes under altered environmental conditions. Here, we present high resolution (50 × 50 nm) elemental spatial distribution in pristine coccoliths of *Coccolithus pelagicus* and *Gephyrocapsa oceanica* reconstructed via X-ray fluorescence analyses at synchrotron. The studied specimens are from control culture and metal-enriched (V, Ni, Zn and Pb) experiments. The analysed specimens produced under stress conditions, display an irregular shape and are thinner, especially in the external rim, with ca. 1/3 lower Ca concentrations compared to specimens from the control. The same specimens also have higher Sr/Ca ratio with highest values in the coccolith external rim, suggesting that difficulty in calcification is additionally reflected in increased Sr/Ca ratios. Selenium is found in the coccolith as possible substitute of carbonate in the calcite. V and Pb apparently did not interact with the coccoliths while Zn and Ni were deposited on the coccolith surface.

## Introduction

**Figure 1:**
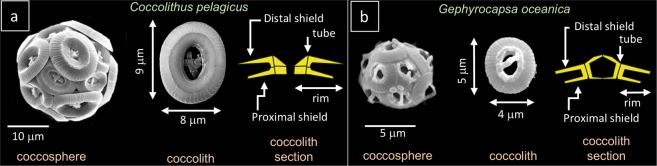
*Coccolithus pelagicus* and *Gephyrocapsa oceanica*. (**a**) Scanning electron microscope (SEM) micrograph of a *Coccolithus pelagicus* coccosphere and coccolith, and schematic cross section of *C. pelagicus* coccolith; (**b**) Scanning electron microscope (SEM) micrograph of a *Gephyrocapsa oceanica* coccosphere and coccolith, and schematic cross section of *G. oceanica* coccolith. SEM pictures are from Faucher *et al*.^11^ experiments.

Coccolithophores are unicellular phytoplanktonic marine algae which produce an exoskeleton made of calcite, called coccosphere (Fig. 1), formed by several single platelets, named coccoliths. Each coccolith is ca. 3 to 10 µm long and it is composed of an assembly of calcite crystals^1^. Although coccolithophores are micrometric in size, they are widespread in all oceans and through photosynthesis and biomineralization, contribute significantly to the long-term sequestration of CO_2_, being responsible for up to 10% of global carbon fixation^2^. As coccolithophores are crucial contributors to ocean biogeochemical cycles, much research has focused on the mechanisms that regulate the calcification process as well as their vital functions^3^. Several works tested the effects of altered carbonate chemistry on calcification indicating that changes in seawater CO_2_ concentration and/or CO_2_-related changes of the carbonate system, can decrease the biocalcification^4–6^, reduce the carbon fixation with production of less calcified or malformed coccoliths and modify phytoplankton species composition e.g.^3,7,8^. Coccolithophore calcification may also be negatively affected by low nutrients and/or biolimiting metal concentrations (e.g., Fe, Zn, Se), showing reduced calcification rates and eventually smaller and/or malformed coccoliths^9–12^. In this regard, coccoliths can be important indicator of the “health state” of the cell because they are produced intracellularly, within the coccolith vesicle^13^ and once formed, they migrate towards the cell margin where they are extruded^14^, and interlock with other coccoliths to form the coccosphere (Fig. 1).

Several studies were performed to reconstruct coccolith size, thickness and mass^15–17^, including X-ray nanotomography^18^. Minor element analyses in coccoliths have become increasingly relevant for both paleo-proxy assessment and the understanding of the mechanisms of calcification. These studies focused on the Mg/Ca and Sr/Ca ratios in coccoliths^19–23^ interpreted to vary with temperature^24,25^ and fertility^21,26^, respectively. Studies were also performed on Ca isotopic fractionation^27^ but very little is known about coccolith elemental concentration^28^, spatial distribution^29^ and partitioning. Obtaining this information is useful to constrain conceptual calcification models and to reconstruct possible dependence on environmental conditions.

In order to gain such information in coccoliths (ca. 3 to 10 µm), X-ray fluorescence (XRF) analyses with synchrotron radiation are ideal since they have a very high spatial resolution and allow to detect and quantify elements present even in trace amounts. Our work treasured the pioneering work of Suchéras‐Marx *et al*.^29^ who analyzed via XRF two Jurassic specimens of *Watznaueria britannica* and obtained their elemental composition and spatial distribution (with a resolution of 100 nm), although partially obscured by clay contamination and diagenesis.

In our study we made a step forward via investigation, in high resolution (50 × 50 nm), of pristine coccoliths of *Coccolithus pelagicus* and *Gephyrocapsa oceanica* from known culture conditions, which included the control (optimal conditions) and metal-enriched (V, Ni, Zn and Pb) experiments.

The questions we wanted to answer to are: (1) Which elements are present in the coccolith calcite? (2) Which is their spatial distribution in the coccolith? (3) Does the calcite composition change when the culture solution composition is altered? (4) Are the spatial distribution and concentration of Ca and Sr in coccoliths a function of the medium conditions? (5) Is there a difference in composition between the calcite of coccoliths belonging to the two tested species? (6) Are the metals (V, Ni, Zn, Pb) added to the culture solution found in the calcite?

The final scope is to understand if there is a “waste bin” effect so that these elements (V, Ni, Zn, Pb) are forced into the calcite as a strategy of the cell to survive toxic concentrations and to detect if metal-enrichments in the culture solution induce any change in coccolith elemental composition.

## Results

### X-ray fluorescence analyses of coccoliths

**Figure 2:**
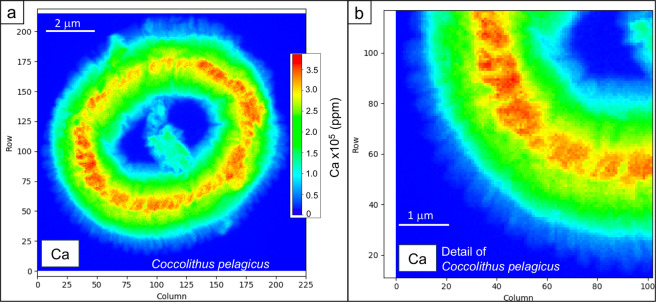
Calcium X-ray fluorescence (XRF) maps of *Coccolithus pelagicus*. **(a**) XRF map showing the Ca concentrations (ppm) and distribution in *Coccolithus pelagicus*. The XRF map is composed of ca. 40k data points each corresponding to a pixel 50 × 50 nm; (**b**) Close up of Ca-XRF map. The single coccolith elements constituting the rim are visible in light blue. The thicker part (in red) corresponds to the coccolith tube.

A total of 7 specimens of *Coccolithus pelagicus* (Fig. 1a) and 2 specimens of *Gephyrocapsa oceanica* (Fig. 1b) were studied by X-ray fluorescence (XRF) analyses performed at the European Synchrotron Radiation Facility (ESRF), Grenoble (France) on the beamline ID16B (see methods). The study was conceived to reconstruct the elemental distribution in high resolution, 50 × 50 nm, necessary to detect and quantify trace elements in specimens which are on average 3 to 10 μm long. Each *C. pelagicus* composition map obtained with this method is composed of 16000 to 35000 data points (Fig. 2a,b) and *G. oceanica* maps are made of 3000 to 6000 data points. The two analyzed species produce placolith-type coccoliths consisting of a central tube which connects a lower proximal shield with an upper distal shield and encloses the central area (Fig. 1a,b). The *C. pelagicus* coccoliths are relatively large (average length of 9 μm) and have a central area spanned by a disjunct bar (Fig. 1a), whereas *G. oceanica* coccoliths are smaller (average length of 5 μm) and have a wide central area spanned by a bridge (Fig. 1b). The specimens analyzed in the current work were taken from the filters of culture experiments of Faucher *et al*.^11^ (see methods). In particular, we selected and isolated *C. pelagicus* coccoliths from control (C) culture grown in artificial seawater (see methods and Supplementary Table 4). *C. pelagicus* specimens were also isolated from cultures with medium (M) and high (H) trace metal (V, Ni, Pb and Zn) concentrations^11^. Specimens of *G. oceanica* were picked only from M and H experiments since specimens from the control culture were no longer available.

**Figure 3:**
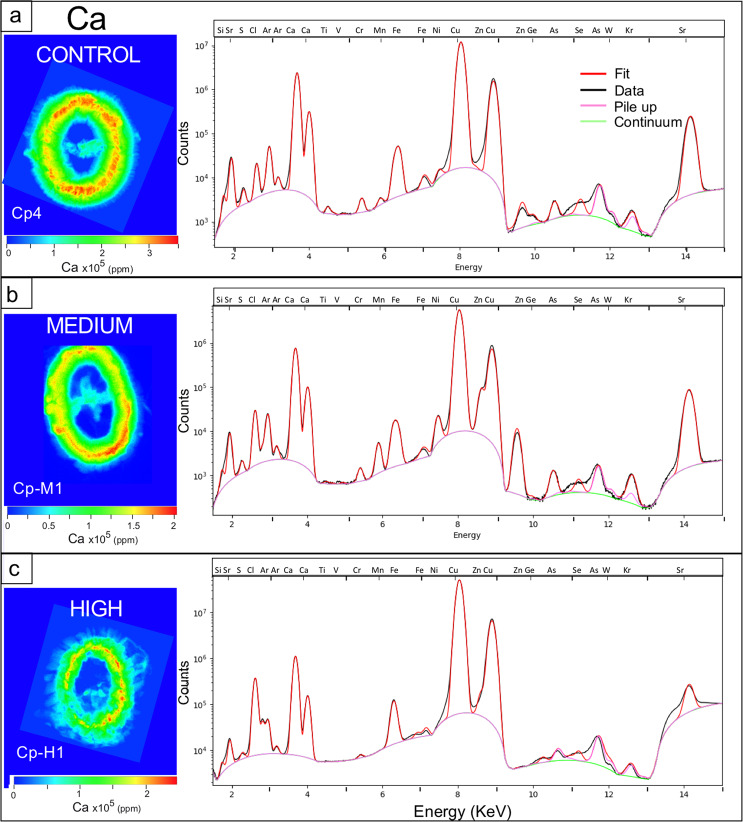
X-ray fluorescence (XRF) spectra of three *Coccolithus pelagicus. Coccolithus pelagicus* specimen cultured under (**a**) control condition (C), (**b)** medium (M) and (**c)** high (H) trace metal (Ni, V, Pb and Zn) concentrations as reported in Faucher *et al*.^11^. The maps of Ca (ppm) have different concentration scales in order to highlight the element distribution. The fits were calculated with PyMCA^30^.

This selection of coccoliths allowed us to investigate pristine specimens that were cultured under known conditions of increasing metal concentrations. For each analyzed coccolith, we obtained a XRF spectrum for each 50 × 50 nm pixel (Fig. 3, Supplementary Fig. 1) reporting several elements comprised between Mg and Sr (K-lines). After fitting with PyMCA^30^, semiquantitative concentration maps were calculated correcting the instrumental and experimental conditions by means of comparison with a standard sample (see methods for details). The pixel by pixel elemental concentrations compose the distribution maps obtained for each studied specimen.

Some of the elements visible in the XRF spectra are strictly related to the experimental hutch (Ar, Kr) or to the detector (Si), while Cu comes from the transmission electron microscope (TEM) support grid. Some of the elements introduced in higher concentrations in M and H experiments are present in low or very low concentrations (Ni and V) in some of the samples, Zn is present only in a few samples (Table 1), and Pb was very difficult to detect (only the low intensity L-lines can be seen with the incident energy used); due to its unreliability, it was decided not to consider Pb in the discussion. Moreover, some of Pb L-lines overlap with As Kalpha-line: what we fit as As may be a combination of the As K-lines and Pb L-lines. In all the maps and the tables, this is reported as As concentration, for the sake of simplicity.

**Table 1:** *Coccolithus pelagicus* and *Gephyrocapsa oceanica* average i/Ca and Ca and Sr concentrations detected via X-ray fluorescence (XRF) in the studied samples.

Experiment	Specimen	Species	N. of data points	Sr/Ca (mmol/mol)	Se/Ca (mmol/mol)	As/Ca (mmol/mol)	Cl/Ca (mmol/mol)	Fe/Ca (mmol/mol)	Zn/Ca (mmol/mol)	Ni/Ca (mmol/mol)	V/Ca (mmol/mol)	Ca (ppm)	Sr (ppm)
Control	Cp4	*C. pelagicus*	30026	3.39	0.045	0.094	124	2.54		0.49		1.65E + 05	1.17E + 03
	Cp7	*C. pelagicus*	20202	4.03	0.049	0.098	103	2.04		0.62		1.28E + 05	1.11E + 03
Medium	Cp-M1	*C. pelagicus*	17574	3.64	0.036	0.091	5493	2.49	7.12	2.75		8.93E + 04	7.04E + 02
	Cp-M2	*C. pelagicus*	35938	5.07	0.043	0.109	619	4.65	5.80	2.96		7.36E + 04	8.70E + 02
High	Cp-H1	*C. pelagicus*	34158	10.10	0.472	0.517	5121	5.20			1.21	6.63E + 04	1.06E + 03
	Cp-H2	*C. pelagicus*	16528	5.10	0.218	0.271	19115	2.22				1.15E + 05	1.12E + 03
	Cp-H6	*C. pelagicus*	7142	5.35	0.127	0.147	2053	2.05			0.61	9.80E + 04	1.05E + 03
Medium	Go-M1	*G. oceanica*	3294	4.45		0.001	1570	3.53				3.74E + 04	3.36E + 02
High	Go-H1	*G. oceanica*	5538	6.31		0.389	1985	3.94				2.82E + 04	3.21E + 02

Specimens are grouped on the basis of the culture experiment (from Faucher *et al*.^11^): control (C) conditions, medium (M) and high (H) V, Ni, Zn and Pb concentrations. As discussed in the text, As may represent the contribution of As K-lines and Pb L-lines. Se/Ca values of Go-M1 and Go-H1 are neglected as the peak spectrum is badly resolved.

Some of the samples have a relatively small V Kalpha peak at about 4.9 keV, which was possible to fit in a reliable way (Table 1, Supplementary Fig. 1). Nickel K-line is at shoulder of one of the very large Cu K-lines: for this reason, its quantification was not straightforward. When in doubt, it was decided not to include the element in the fit. The latter was used as a general rule in this work: when there was no certain evidence of the presence of the elements, they were left out of the fit, so that there was no risk of overfitting the spectra (Supplementary Fig. 1).

Mn, Ti and Cr are randomly found in traces in most of the studied coccoliths but since they are localized in very small areas (Supplementary Fig. 2), no further discussion is made on these elements. However, probably they derive from the culture solution of Faucher *et al*.^11^ who added trace elements following Guillard *et al*.^31^ (see methods). W is also concentrated in few spots (Supplementary Fig. 2) which are compatible with contamination from the tungsten needle used for coccolith picking.

### Elements detected in the coccolith calcite

**Figure 4:**
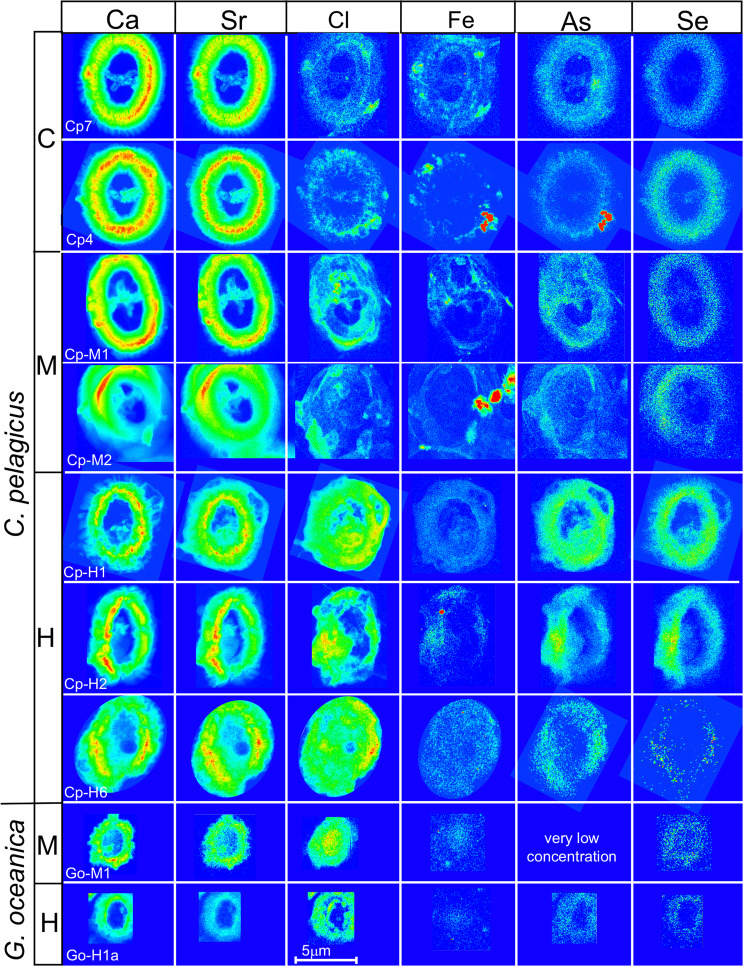
X-ray fluorescence (XRF) maps showing the distribution of Ca, Sr, Cl, Fe, As and Se in *Coccolithus pelagicus* and *Gephyrocapsa oceanica*. Specimens cultured under control conditions are indicated with “C”, while “M” and “H” refer to specimens cultured under medium (M) and high (H) trace metal (Ni, V, Pb and Zn) concentrations as reported in Faucher *et al*.^11^. The maps have different concentration scales in order to highlight the element distribution. Note that specimen Cp-M2 and Cp-H2 are partially tilted. Arsenic maps may represent the contribution of As K-lines and Pb L-lines as discussed in the text.

The high-resolution approach of the analyses allowed to obtain detailed elemental maps showing the spatial distribution of concentrations in the specimens. The Ca analyses are extremely informative: they show the coccolith structures, morphologies and thickness (Figs. 2–4). Ca concentrations are proportional to the calcite amount in the coccolith; consequently, the red parts in the maps (Figs. 2–4) correspond to the thicker portions of the coccolith coinciding with the tube, while the light blue colors represent the thinner part of the coccolith rim where single calcite crystals composing the shields are visible (Figs. 2b, 3 and 4). Comparison of the Ca maps of the same species from different experiments (i.e., different metal concentrations in the culture) reveals that the analysed *C. pelagicus* coccoliths from M and H experiments have irregular morphology and outline and the central bar in H specimens is missing (Fig. 4). In the two *G. oceanica* specimens from M and H experiments the bridge is absent, and the outline is also irregular (Fig. 4). Specimens with these features were also observed in scanning electron microscope (SEM) pictures of coccoliths from the same experiments (Supplementary Fig. 3).

**Figure 5:**
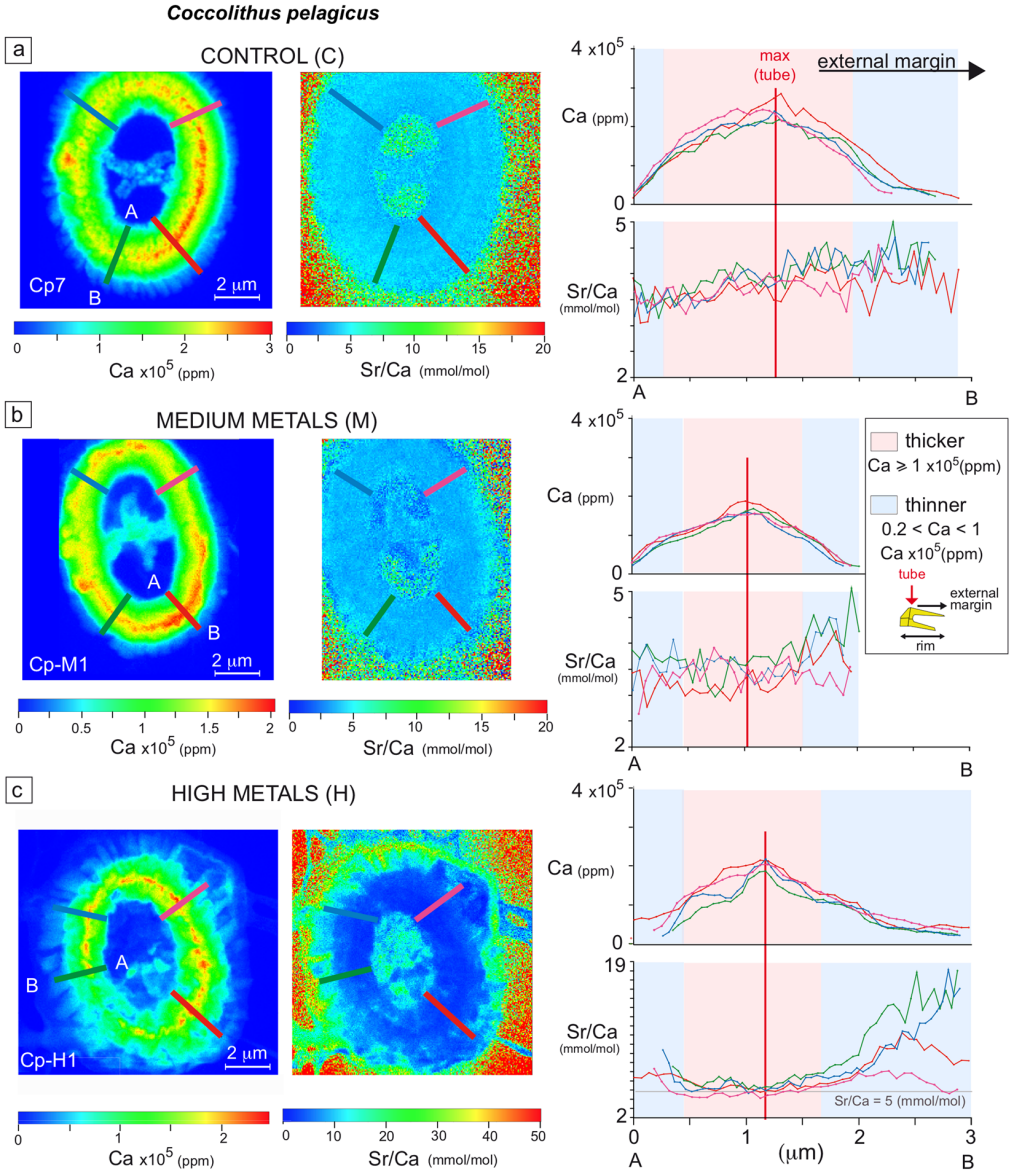
Ca and Sr/Ca profiles across the *Coccolithus pelagicus* coccolith rim. On the left X-ray fluorescence (XRF) maps are reported for Ca concentrations and Sr/Ca ratios in the three *Coccolithus pelagicus* specimens. The specimens are from (**a**) control (Cp7), (**b**) medium (Cp-M1) and (**c**) high (Cp-H2) metal experiments^11^. In each map four transects are traced in different colors. Ca (ppm) and Sr/Ca (mmol/mol) profiles are reported on the right. The light blue pattern in the transects highlights the regions of the profile where 0.2 × 10^5^ < Ca < 1 × 10^5^ (ppm), the red pattern indicates the regions of Ca ≥ 1 × 10^5^ (ppm). The background from the membrane is excluded from the transects. Considering the Ca concentration to be proportional to the coccolith thickness, the profiles provide indication of the lateral change in coccolith thickness. The Sr/Ca profiles highlight higher mean values in the specimen form H experiment and higher average Sr/Ca values in the external part of the margin of all specimens.

The total average Ca concentration (ppm) in *C. pelagicus* from M and H experiments is ca. 1/3 lower than the average Ca concentration detected in specimens from C culture. The *G. oceanica* specimen from H experiment has lower mean Ca concentration with respect to the coccolith from M culture (Table 1). The Ca/coccolith area (ppm/μm^2^) is higher in *C. pelagicus* specimens from C culture compared to specimens from M and H experiments and *G. oceanica* has moderately higher Ca/coccolith area (ppm/μm^2^) in the specimen from M experiment compared to the specimen from H experiment (Supplementary Fig. 4). This suggests that the studied *C. pelagicus* coccoliths from H experiments are thinner than the coccoliths from C culture. No major difference in thickness of *G. oceanica* coccoliths from M and H experiments is evidenced. More detailed information about Ca concentrations can be obtained by looking at the Ca spatial distribution across the shield profiles which reflect the morphology of the coccolith and its thickness (Fig. 5, Supplementary Fig. 5). The Ca profiles across the same coccolith are similar except for minor differences in specimens from H experiment due to coccolith irregular shape. The profiles show that *C. pelagicus* coccoliths from C culture have a portion of the coccolith with Ca concentrations above 1 × 10^5^ ppm which is more extended than in specimens from M and H experiments (Fig. 5, Supplementary Fig. 5). The specimens from C culture also show the highest Ca values in correspondence of the tube. These evidences are interpreted to reflect thicker *C. pelagicus* coccoliths from C culture compared to coccoliths from M and H experiments which are thinner especially in the external rim. Also *G. oceanica* from H experiment shows moderately lower Ca values in the tube compared to the specimen from M experiment (Supplementary Fig. 5).

The Sr maps show a distribution of the element which is very similar to that of Ca (Fig. 4). The two elements are strongly correlated (average r = 0.98, Supplementary Table 2) and show almost identical profiles in cross sections (Fig. 5, Supplementary Fig. 5). This confirms the occurrence of Sr in the coccolith calcite structure as widely known^19,20,22,23^. In this work, Sr/Ca ratios are reconstructed on a thousand of data points for each pristine coccolith and allow to accurately appreciate the variations in the Sr/Ca ratio with respect to the coccolith morphology. The *C. pelagicus* and *G. oceanica* coccoliths from H experiments have average Sr/Ca ratios which are higher than in M and C (for *C. pelagicus*) specimens. Moreover, the transects show that Sr/Ca ratios increase significantly towards the coccolith external rim (Fig. 5, Supplementary Fig. 5). Since Sr/Ca ratios are not reliable where Ca concentrations are very low, the transects and further calculations do not consider the regions where Ca values are below 0.2 × 10^5^ (ppm) including the very end of the coccolith external rim and the background. Coccolith Sr/Ca ratios do not correlate with Ca concentrations (Supplementary Table 3) suggesting that Sr/Ca ratios are mostly independent from thickness. This is also visible in the transects, where regions with equal Ca concentrations from the internal and the external rim of the coccolith, show different Sr/Ca values, always higher in the external rim (Fig. 5, Supplementary Fig. 5).

**Figure 6:**
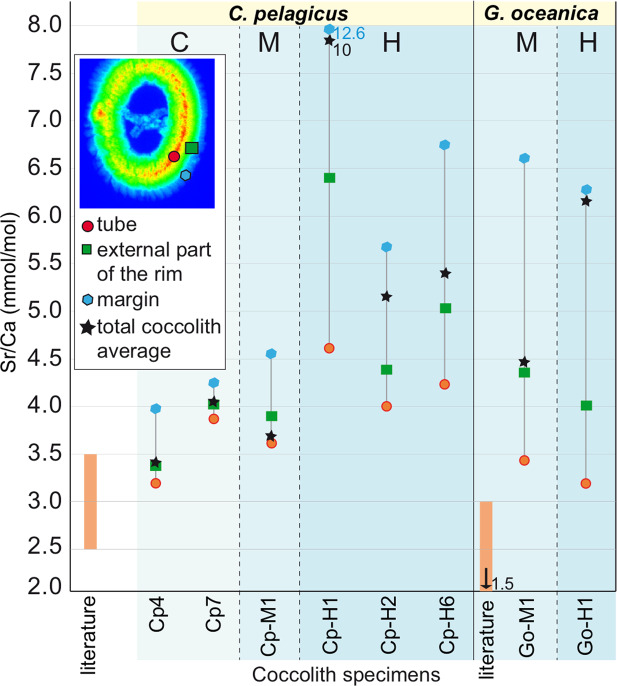
Sr/Ca ratios (mmol/mol) reported for each studied specimen. Average Sr/Ca ratio (expressed in mmol/mol) based on at least 30 data points from the tube of the coccolith (red dots), the external part of the rim (green squares) and the rim margin (blue hexagons). Black stars represent the average Sr/Ca ratio of the whole coccolith. Specimens are divided per experiment: C = control, M = medium metal concentrations, H = high metal concentrations following Faucher *et al*.^11^. Literature Sr/Ca ranges for *C. pelagicus*^21^ and *G. oceanica*^27,47,48^ are reported in orange. *Coccolithus pelagicus* specimen Cp-M2 is excluded from the representation since it is tilted, and it is not possible to precisely identify the tube, rim and margin.

In order to better estimate the lateral variations through the rim, the average Sr/Ca values were calculated for three regions of the coccoliths (considering at least 30 data points for each region): (i) the tube (red in Fig. 6), (ii) the external rim (green in Fig. 6), and (iii) the coccolith margin (light blue in Fig. 6). The results are expressed as mmol/mol for comparison with literature data and show a progressive increase in the Sr/Ca ratio going from the thicker part of all studied coccoliths (tube) towards the margin (Fig. 6). This increase is less marked in *C. pelagicus* coccoliths from C and M experiments compared to coccoliths from the H experiment, characterized by much higher Sr/Ca values in the margin. In *G. oceanica* both specimens from M and H experiments have higher Sr/Ca ratio in the margin. Among the two studied species from H experiment, *G. oceanica* has lower Sr/Ca in the tube while the Sr/Ca ratios of the rim are closer to those of *C. pelagicus*.

Taken together, these results indicate that *C. pelagicus* and *G. oceanica* coccolithophores exposed to metal-enriched culture solution, produced smaller (as indicated by Faucher *et al*.^11^), irregular and possibly thinner coccoliths, especially in the external part of the rim. These coccoliths also displayed a larger Sr/Ca ratio, particularly in the margin, with a relatively higher variability within specimens of the same species cultured under stress conditions.

**Figure 7:**
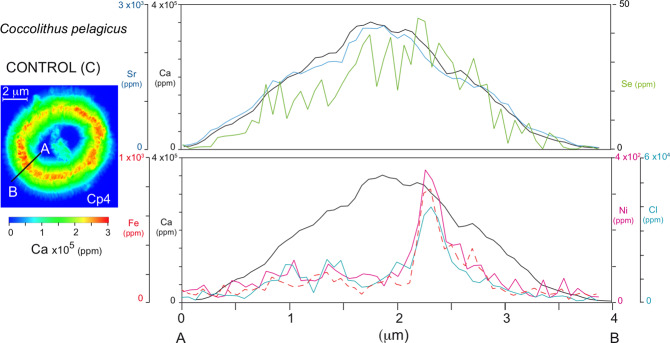
Ca, Sr, Se, Ni, Fe, and Cl profiles across *Coccolithus pelagicus* coccolith rim. On the left the X-ray fluorescence (XRF) map show the Ca concentration (ppm) and distribution in one *Coccolithus pelagicus* specimens from the control experiment (Cp4). The transect “A-B” across the coccolith rim is reported in the XRF map. The upper profile on the right reports Ca (black), Sr (blue) and Se (green). The lower profile represents Ca (black), Cl (light blue) and the elements with a trend similar to Cl such as Fe (dashed red) and Ni (pink).

The XRF analyses evidenced the presence of Se in all *C. pelagicus* and *G. oceanica* coccoliths (Table 1). Selenium was added in the culture medium as a micro-nutrient for the cell (see the methods). Selenium distribution mimics the coccolith shape (Fig. 4) however, Se Kalpha peak at 11.2 keV in Go-M1 and Go-H1 spectra is badly resolved to trust Se/Ca values (Supplementary Fig. 1). The average Se/Ca ratios are relatively similar (ca. 0.04 mmol/mol) in all studied *C. pelagicus* from the C and M experiments (Table 1), while average Se/Ca ratio is higher in coccoliths from H experiment (Se/Ca = 0.26 mmol/mol) and, in these specimens, Se shows a positive correlation with Ca (r = ca. 0.7, Supplementary Table 2). In all studied specimens Se distribution pattern in cross section is similar to the one of Ca (Fig. 7, Supplementary Fig. 6). On the basis of these results, we infer that Se is incorporated in the coccolith calcite. This is plausible due to its chemical affinity and atomic radius^32,33^. A possible mechanism for selenium in inorganic calcite is the structural incorporation of selenite (SeO_3_^2−^) into calcite (CaCO_3_) by the substitution of carbonate^34^.

### Elements deposited on the coccolith surface

Chlorine is present in all specimens and it is distributed over the coccoliths (Fig. 4). It shows Cl/Ca ratios comprised between 100 and 19120 mmol/mol (Table 1). The Cl/Ca progressively increases from *C. pelagicus* specimens of C culture to specimens from the H experiment as well as in *G. oceanica* from M to H culture (Table 1). The Cl pattern in the coccolith cross sections (light blue in Fig. 7) differs from that of Ca and no correlation exists between Cl and Ca (Supplementary Table 2). Cl is very abundant in the solution^11^ as well as in the coccolithophore cell plasma^35^. The spatial distribution of Cl in the studied specimens suggests that Cl is on the coccolith surface.

The analyses also highlight the presence, in all *C. pelagicus* specimens, of Fe distributed over the coccoliths and, in some specimens, is locally enriched in very small areas (Fig. 4). The average Fe/Ca ratio is comprised between 2 and 5.2 mmol/mol. In *G. oceanica*, Fe maps are less defined, but average Fe/Ca is similar to that of *C. pelagicus*. Since there is no correlation between Fe and Ca and the Fe profile in the transects shows a trend which is similar to Cl rather than Ca (Fig. 7, Supplementary Fig. 6), we exclude that Fe is incorporated into the calcite. Fe is here interpreted to be on the coccolith surface.

**Figure 8:**
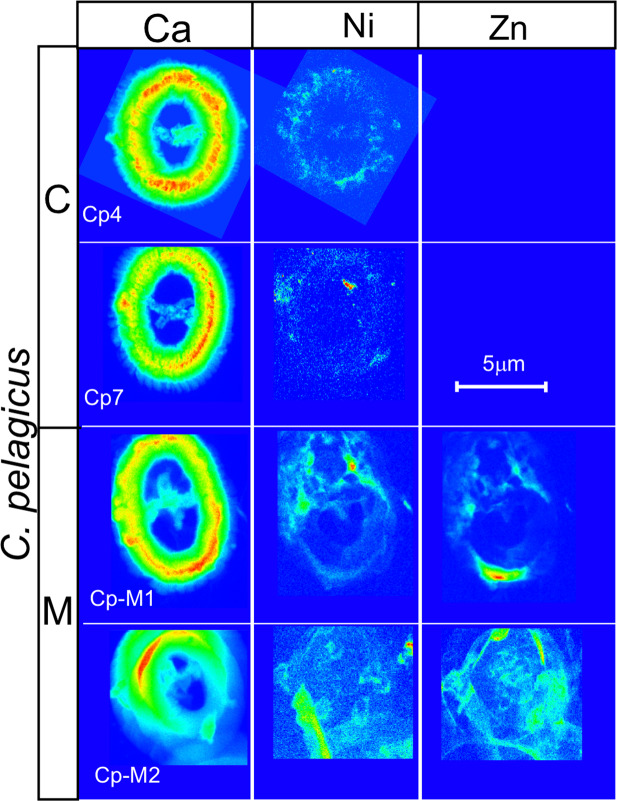
X-ray fluorescence (XRF) maps of Zn and Ni in *Coccolithus pelagicus*. For each specimen the sample number and the experiment are reported. Control conditions (C) and medium (M) metal (Ni, V, Pb and Zn) concentrations as indicated in Faucher *et al*.^11^.

Out of the four elements V, Ni, Zn and Pb, introduced in higher concentrations in the M and H experiments^11^, V is absent in most samples except in Cp-H1 (average V/Ca = 1.21 mmol/mol) and Cp-H6 (average V/Ca = 0.61 mmol/mol) where it is found in very low abundances (Table 1) with flat distribution all over the studied area, Pb is not easily detected as explained above, and Ni and Zn are absent in *G. oceanica* specimens. Ni is present (average Ni/Ca = 0.55 mmol/mol) in *C. pelagicus* coccoliths from C culture and M experiment (average Ni/Ca = 2.8 mmol/mol) and Zn (average Zn/Ca = 6.4 mmol/mol) is found only in *C. pelagicus* from M culture (Table 1). Ni and Zn are not homogenously distributed on the analysed coccoliths, showing localized enrichments (Fig. 8). Zn, Ni and Fe do not display a correlation with Ca (Supplementary Table 2) and their profiles in coccolith sections are similar to the Cl profile (Fig. 7, Supplementary Fig. 6). The distribution patterns as well as the correlation with Cl, suggest that these elements are not incorporated in the calcite structure.

## Discussion

The XRF analyses performed at ESRF (ID16B) with a 50 × 50 nm resolution, show that the method applied provides very detailed information on the presence/absence and spatial distribution of the elements with respect to the coccolith calcite and their relationships. The method allows to gain thousands of datapoints for each specimen and, consequently, to see how the individual element changes in concentration relative to the coccolith morphology, size and thickness.

The composition of *C. pelagicus* coccoliths cultured under C conditions, shows that Se was incorporated in the structure of coccolith calcite, in addition to Sr. Selenium is present in traces (i/Ca <0.5 mmol/mol) in all studied coccoliths. This is the first evidence of Se in pristine coccoliths and opens the question on the mechanisms involved in its incorporation into the calcite structure. The importance of Se for the functioning of the cell is well documented^36^. Selenium is a nutrient stimulating coccolithophore growth^36,37^. We hypothesize that Se reaches the coccolith vesicle exchanging with S which is an important element in coccolithogenesis being one of the components of the sulfated polysaccharides involved in the shaping of the coccolith calcite crystals^38,39^. Once Se, is in the coccolith vesicle, it may interreact with the forming coccolith where selenite (SeO_3_^2−^) substitutes the carbonate and enters the calcite structure^32–34^. The partitioning coefficient of Se (D_Se_), calculated for the studied coccoliths from C culture (see methods), is 0.048. The D_Se_ of *C. pelagicus* coccoliths from the C culture is ca. two times higher than the D_Se_ of inorganic calcite = 0.02 ± 0.01^34^ and increases ca. four times in the specimens cultured under H experiment. These data are in favor of a biological influence in the incorporation of Se in the coccolith calcite.

Our work also shows that coccolith size reduction (documented in Faucher *et al*.^11^), is not the only effect of higher metal concentrations, but, as far as the analyzed specimens are concerned, they have an irregular shape and they are thinner. The major novelty is about coccolith thickness as we attested that the average amount of Ca in coccoliths from higher metal experiments, is ca. 1/3 lower than the average Ca concentrations in specimens from the control (Table 1). In the last decades, the measurement of coccolith thickness has been matter of investigation for the calculation of coccolith mass as an indicator of current and past ocean carbonate chemistry^16,18,40–43^. A positive correlation between coccolith size and cell size occurs within and between species^18,41,44^ and the same accounts for coccolith thickness^18,43^. It has been documented that the amount of calcite per cell surface area varies with cell size and small cells are characterized by thinner coccoliths^18^. *C. pelagicus* and *G. oceanica* from the M and H experiment were affected by size reduction of the coccosphere and cell diameter^11^. The reduction in coccolith thickness detected here further points to a reduced calcification. To date, variations in coccolith mass and/or thickness were mainly evidenced in response to changes in surface water carbonate chemistry^6,42,45^ and productivity^46^. Our results have important implications related to the negative effects of higher metal concentrations on calcification. The thinning of coccoliths summed to smaller mean size^11^, implies that less CO_2_ is incorporated by *C. pelagicus* and *G. oceanica* during calcification. This may have a large impact on the ocean-atmosphere system although it is the proportion between the production of organic carbon and total calcification which determines the feedbacks on climate and atmospheric carbon dioxide concentrations^3^.

New insights also come from the Sr/Ca ratio of the studied specimens. Sr/Ca determinations of coccolith calcite has been the objective of several studies either on sediment fractions, monospecific cultures and single specimens. These studies highlighted that the average Sr/Ca ratio of coccoliths, is higher than in abiogenic calcite (0.13 mmol/mol^21^) as the distribution and partitioning of Sr are driven by a combination of precipitation kinetics and cellular physiology^25,26,47^.

Here, Sr/Ca values are measured, for the first time, on a large number of data points for the same specimen and, although the analytical method is different from those applied in dedicated works^20,21,25,47^, the average Sr/Ca of *C. pelagicus* specimens from the control (3.7 mmol/mol) is consistent with the average Sr/Ca ratio (3 mmol/mol) reported in literature^21,26^. In *C. pelagicus* coccoliths from C culture, the distribution pattern of Sr/Ca is also relatively homogenous, displaying a minor discard between the tube and the external margin. The average Sr partitioning coefficient (D_Sr_) in studied *C. pelagicus* from C culture is 0.42 which is also close to the average reference values^21^. Differences, with respect to the specimens from C culture and the literature values, occur in *C. pelagicus* specimens from metal-enriched H experiment characterized by ca. double average Sr/Ca ratios (6 mmol/mol) and high Sr/Ca variability among single specimens. These coccoliths also display increasing Sr/Ca ratios through the margin, up to 5.7 and 12.6 mmol/mol in the external margin (Fig. 6). The average D_Sr_ in specimens from M experiment is 0.49 and from H experiment is 0.78. Similarly, *G. oceanica* from M and H experiments have average Sr/Ca ratios (4.4 in M and 6.3 in H coccoliths) and D_Sr_ (0.5 in M and 0.72 in H coccoliths) which are above literature values for specimens cultured in control medium (Sr/Ca = 1.2 to 3 mmol/mol and D_Sr_ = 0.26^48^).

In the literature higher coccolith Sr/Ca ratios have been reported to correlate with higher growth- and calcification rates if the latter were changed by changing macro-nutrient concentrations^49^. If, by contrast, changes in growth- and calcification rates were due to changes in light intensity, no correlation between Sr/Ca ratios and growth- and calcification rates were observed^25,26^. The latter authors therefore concluded that growth- and calcification rates per se do not affect Sr partitioning. This conclusion is supported by our own data which show a negative correlation between Sr/Ca ratios and growth rates. Relatively high Sr/Ca ratios are not unusual for some coccolithophore species. For example, *Scyphosphaera apsteinii* has Sr/Ca ratio around 22.1 mmol/mol in control medium and D_Sr_ = 2.5^48^. To explain high Sr/Ca ratios in *S. apsteinii*, Hermoso *et al*.^48^ proposed that there may be a return flux from the coccolith vesicle to the cytosol leading to a leakage of Ca^2+^ and, consequently a Sr^2+^ enrichment in the coccolith vesicle. The data in our possess do not allow to bring any hypothesis further. The evidence of changes in the Sr/Ca ratios and D_Sr_ in species exposed to a different stress from those investigated so far, opens to more questions regarding the control mechanisms of Sr and Ca incorporation in coccolithophore cells under stress conditions.

Regarding the transition and post-transition metals (Ni, Zn, Pb and V) one of our questions was if these elements could be forced into coccoliths as a detoxification function. For example, in another fossil group, such as planktonic foraminifera, Zn has been detected in the shell of specimens collected from Zn contaminated sea areas as a consequence of detoxing^50,51^. In our study, V is absent in most of the coccoliths and, in the two specimens where it is detected, it is homogenously distributed over the specimens and the background in low concentrations, thus no further interpretations can be made except concluding that V did not interact with the coccolith. Pb is difficult to detect, and Ni and Zn are only present on some *C. pelagicus* coccoliths. In addition to Ni and Zn, Fe is also found in all coccoliths with a similar distribution pattern. Fe was introduced with the same concentration in all experiments (see Supplementary Table 4). The interaction of the transition metals with the coccolithophore cells is known: Zn, Ni and Fe have important biological functions, since they are involved in the cell metabolism and influence the growth^10^. Significant changes in the concentrations of these biolimiting metals alter the biocalcification process and the cell growth rates^10,11,52^. However, there is no evidence of Fe, Zn and Ni in/on pristine coccoliths in literature. The only documentation is in the work of Suchéras‐Marx *et al.*^29^ who found Zn and Fe in *W. britannica* coccoliths but interpreted it to be derived from clay contamination. Our data do not show a clear evidence of an incorporation of Zn and Ni in the calcite but rather their presence on the coccolith surface. The same accounts for Fe. In the studied coccoliths, Fe, Ni and Zn have a distribution that mimics the one of Cl. Cl is part of the cell cytosol and Zn, Ni and Fe have been found in coccolithophore cells^53,54^. This leads to the hypothesis that, in the studied coccoliths, Zn, Ni and Fe are related to the organic matter.

In conclusion, our study does not show clear evidence of a “waste bin” effect of toxic elements in *C. pelagicus* and *G. oceanica*. On the contrary, there is clear evidence that Se enters the coccolith calcite structure. Ultimately, higher metal concentrations induced variations in thickness and in Sr/Ca ratios in the studied coccoliths. This work poses the bases for new studies testing the elemental distribution in other species and larger numbers of specimens for better statistics. Also, other elements not identified here (since ad hoc preparations and different settings are required) will be investigated to have a more comprehensive characterization of coccolithophore calcification and their application as environmental tracers. Since coccolithogenesis process involves complex sequences of cellular and molecular reaction steps and mechanisms, the investigation of coccolith size, composition and morphology, should go along with the studies of the biology and physiology of coccolithophores to understand the effects of perturbed environmental conditions on biocalcification.

## Methods

### Cultures

In this study we analysed specimens of *Gephyrocapsa oceanica* and *Coccolithus pelagicus* from the experiments of Faucher *et al*.^11^ where details are reported. Monospecific cultures of the coccolithophores *G. oceanica* (strain RCC 1303) and *C. pelagicus* (strain PLY182G) were grown as batch cultures in artificial seawater enriched for trace metals^31^ and added with the trace metal chelator (ethylenediaminetetraacetic acid) which guarantees a constant level of bioavailable trace metals for phytoplankton and prevent metal precipitation. The specimens studied in the current work were selected from control (C) cultures as well as from cultures with medium (M) and high (H) concentrations of Pb, Zn, Ni and V (see Supplementary Table 4). Coccoliths were taken when cells were still in their exponential growth phase when cell numbers were low enough to avoid a strong change in the chemical conditions of the growth medium. Scanning electron microscopy (SEM) pictures were taken of several specimens from the same filters where the studied coccoliths were collected. The SEM pictures were captured at the Earth Sciences Department of the University of Milan with SEM Cambridge Stereoscan 360 (Supplementary Fig. 3).

### Coccolith picking and specimen selection

An aliquot of coccoliths from filters form Faucher *et al*.^11^ was suspended and rinsed two times in MQ water for cleaning and then pipetted on a glass slide for picking of single specimens. Picking of coccoliths was performed following a picking method modified from Suchéras *et al*.^55^: individual coccoliths were picked by hand, using a tungsten needle (as suggested by Stoll & Shimizu^56^) with a tip radius < 0.5μm, from the smear slide. After picking, coccoliths were deposited on a 500-nm-thick Cu transmission electron microscope (TEM) grid with amorphous holey carbon film (TAAB) using a Leica DM2700P microscope at 1250 X magnification. Specimens were deposited using a droplet of MQ water to detach the coccolith from the tungsten needle by surface tension.

The partition coefficients are reported with the symbol D_x_ (x stands for the element of interest) and are calculated as the ratio between the molar concentration of the element of interest in the calcite and its molar concentration in the solution^25^.

### X-ray fluorescence analyses

X-ray fluorescence measurements were performed at the European Synchrotron Radiation Facility (ESRF), Grenoble, France, at the beamline ID16B. The beamline is one of the long ones @ESRF, and allows the analysis in 2D and 3D, with a nominal minimum beam size of 25 × 25 nm, with the pink beam (from 17 to 30 keV), and slightly larger (100 × 100 nm) for the lower energies. In our experiment, the incident X-ray beam (pink beam) energy used was 17.1 keV, that allows to measure from Mg (Kalpha = 1.253 keV) up to Sr (Kalpha = 14.098 keV). The applied X-rays beam size was 50 × 50 nm (only Cp-H6 was measured with a lower resolution of 100 × 100 nm). The detector used during the experiment was one 7-elements Si drift detector (see the beamline website for more details). Coccoliths were analysed with 200 ms dwell time per pixel. XRF maps were obtained by scanning line after line, and the corresponding XRF-spectrum (supplementary Fig. 1) was fitted using PyMCA 5.7.0^30^ software. The semiquantitative concentration of each element was calculated correcting the instrumental and experimental conditions by means of comparison with a standard sample: elemental mapping of the NIST 1577b bovine liver standard was used. The standard was positioned on a matrix of Si_3_N_4_ with a thickness of 200 nm. The mass fraction of various elements was checked during calibration (Pb, La, Pd, Mo, Cu, Fe, and Ca), taking into account the effect of the matrix: the instrumental parameters (flux and active area) were refined to obtain concentration values that were within the correct range (taken from the values certified by NIST). Once the concentration values were calibrated, the values for flux and active area were kept fixed, and then used for the evaluation of the elemental concentration in the coccolith samples. The detection limits of K-lines of the elements analysed are, according to the beamline database, of the order of 1–10 ppm with the pink beam (17.1 keV) in biological samples, with an integration time of 0.1 s. There is no information on inorganic samples, but there is no reason to believe for their detection limits to be much worse than those for organic ones, taking also into account that the integration time of the analyses here reported is larger (0.2 s).

## Supplementary information

Supplementary Information.

## Data Availability

All data supporting the findings of this study are available on Zenodo (DOI 10.5281/zenodo.3737571), under Creative Commons Licence (International).
