# Supplementary material for: High resolution spatial analyses of trace elements in coccoliths reveal new insights into element incorporation in coccolithophore calcite
Source: Sci Rep. 2020 Jun 17;10:9825. doi: 10.1038/s41598-020-66503-x (PMC7299995; doi:10.1038/s41598-020-66503-x)
Supplement: Supplementary file 1 — Supplementary Information. [file 41598_2020_66503_MOESM1_ESM.pdf]

Supplementary Information

"High resolution spatial analyses of trace elements in coccoliths reveal new insights into element incorporation in coccolithophore calcite"

Cinzia Bottini[1]*, Monica Dapiaggi[1], Elisabetta Erba[1], Giulia Faucher[1], Nicola Rotiroti[1]

[1]Università degli Studi di Milano, Dipartimento di Scienze della Terra, Milano 20133 Italy

CP7

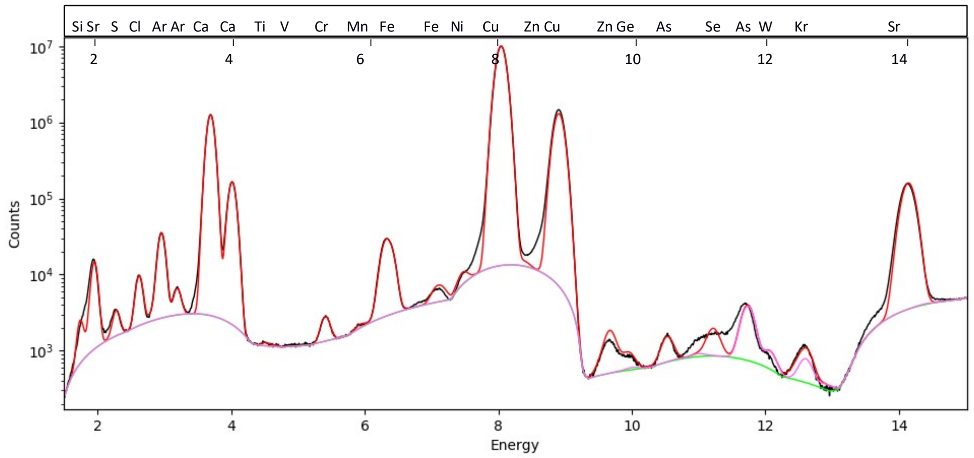

CP4

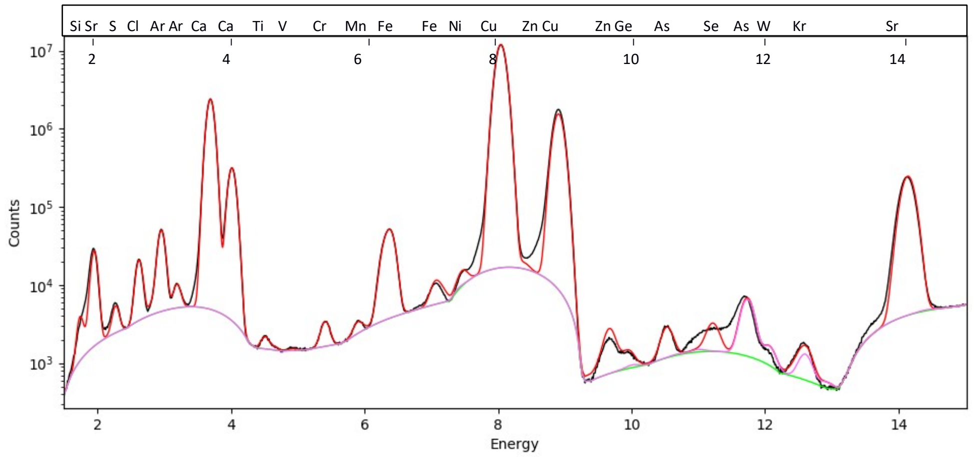

CP-M1

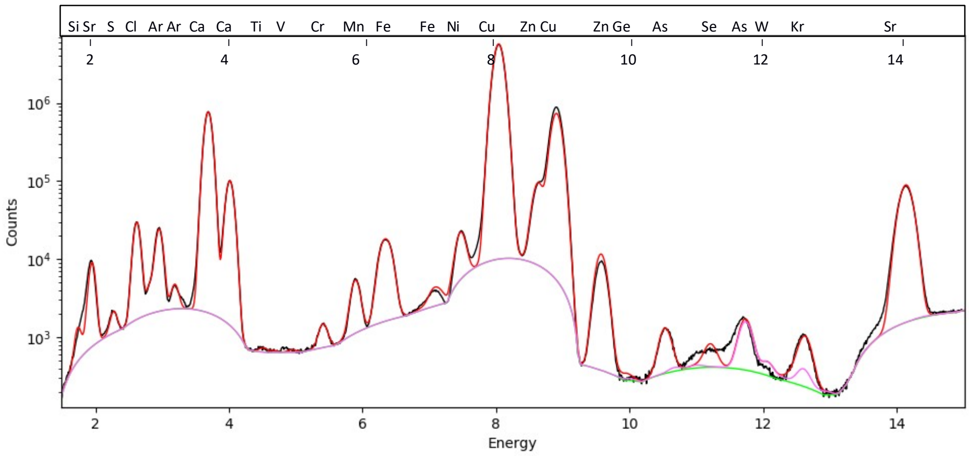

CP-M2

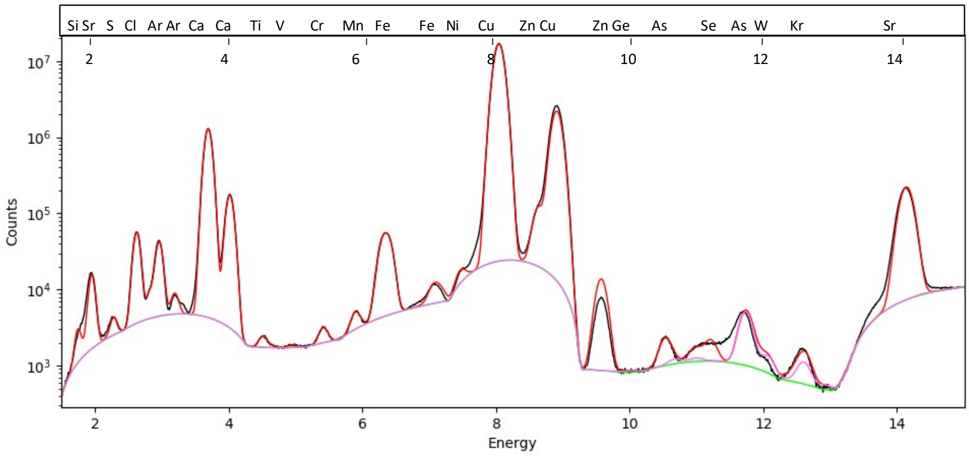

CP-H1

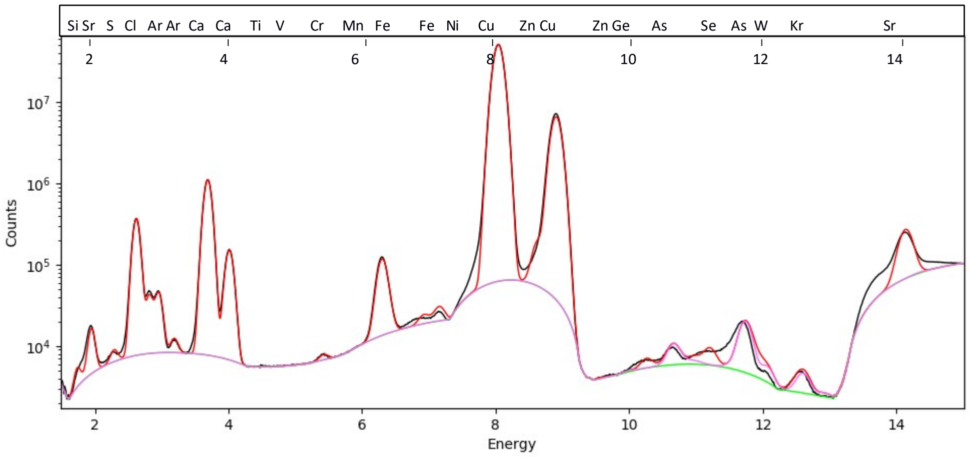

CP-H2

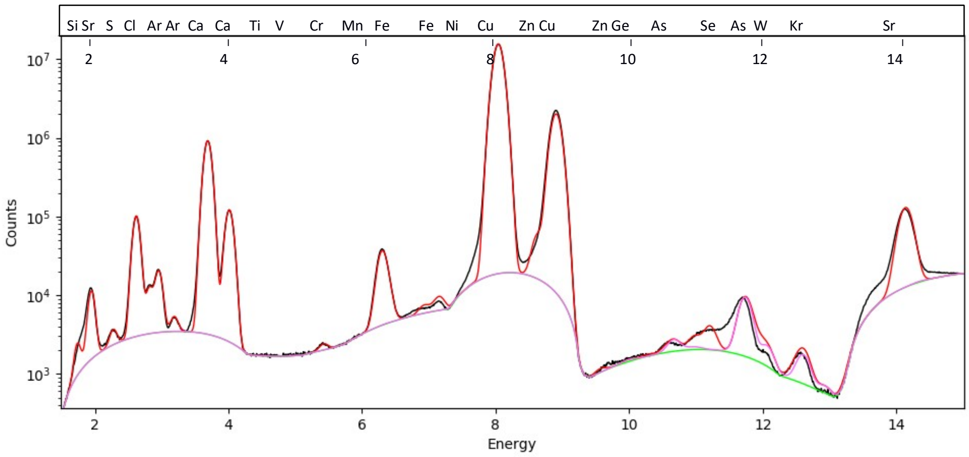

CP-H6

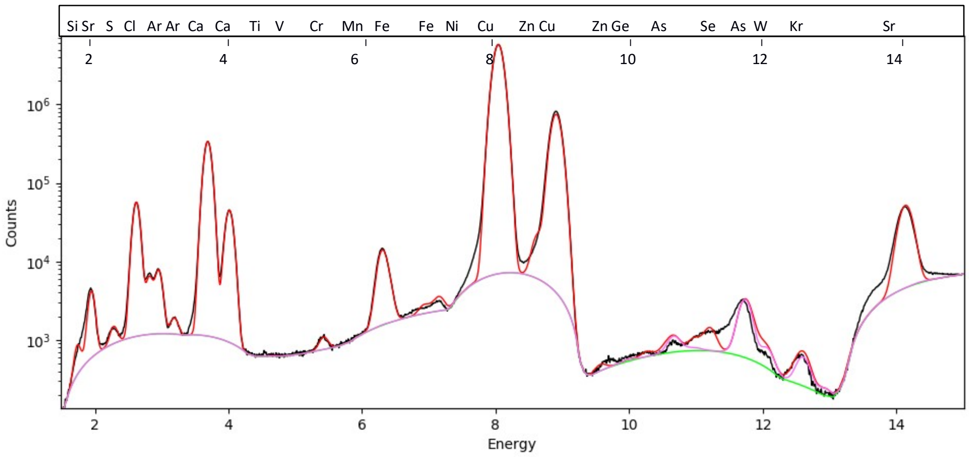

GO-M1

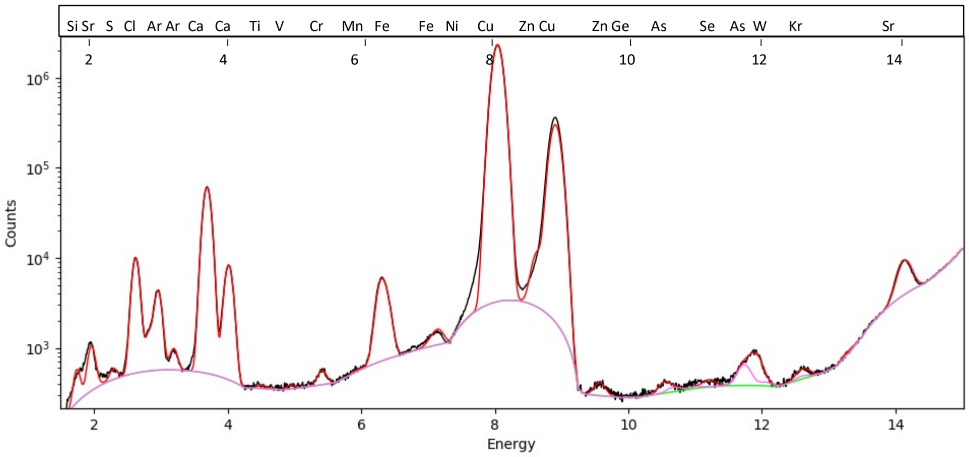

GO-H1

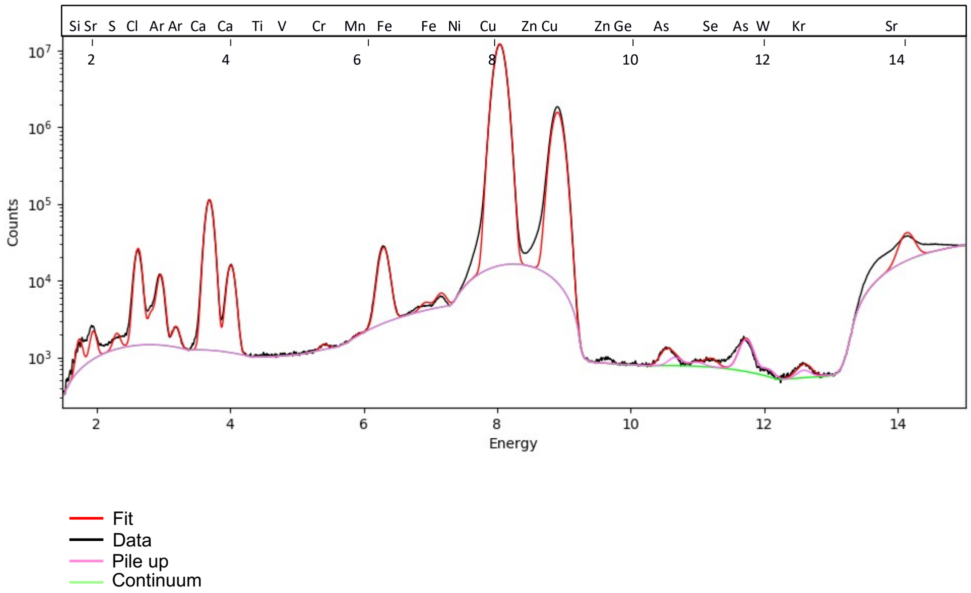

Supplementary Figure_1. X-ray fluorescence (XRF) spectra obtained for the coccolith specimens analysed in this study. The fits are calculated with PyMCA[1] and used to derive i/Ca in Table 1 and Supplementary Table 1. Cp = *Coccolithus pelagicus,* Go = *Gephyrocapsa oceanica*. Remarks: In Cp-H1 sample, the bump at 10.26 keV was fitted with Ga (K-beta) although the peak is not well resolved and, consequently, it is not discussed in the text.

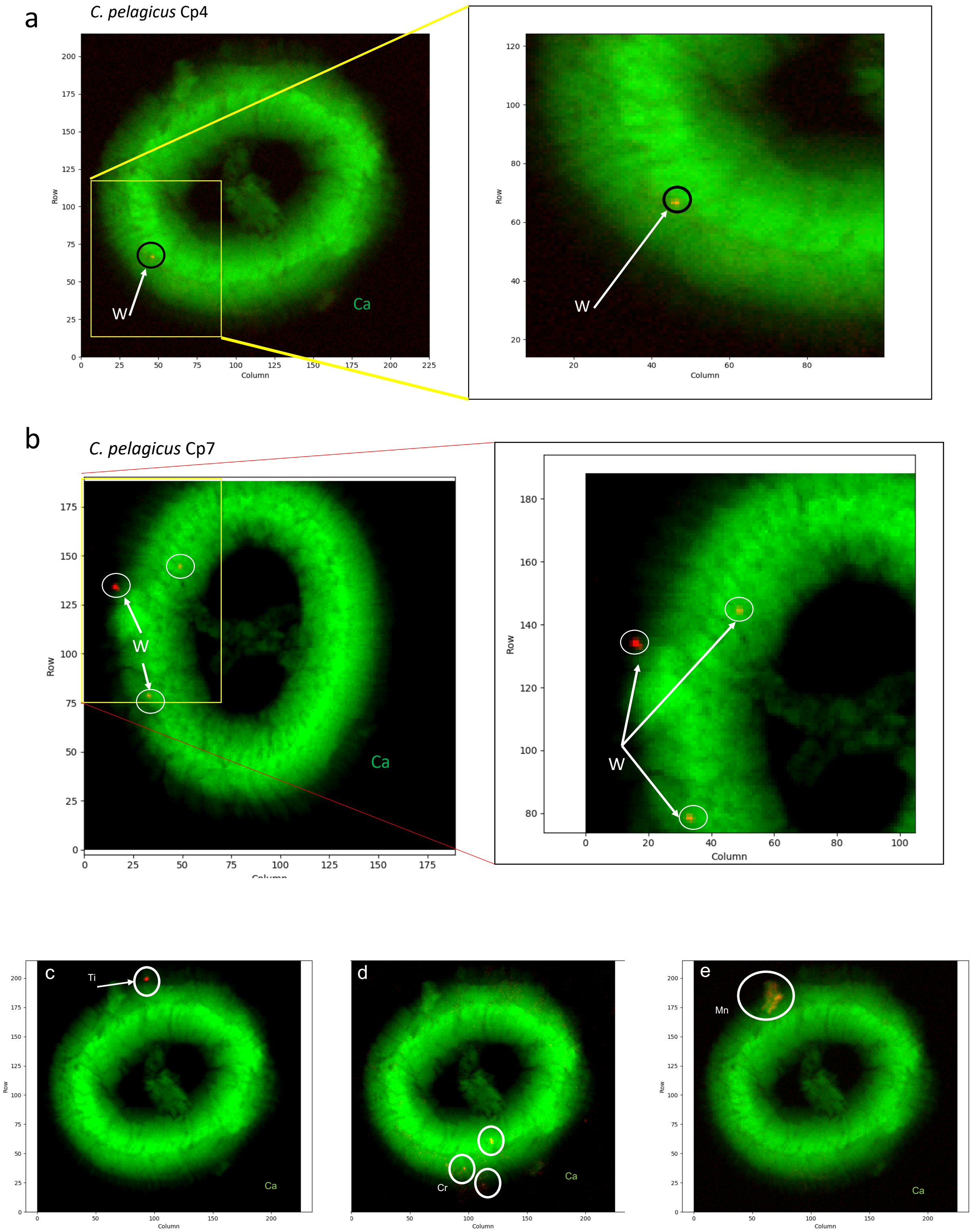

Supplementary Figure_2. X-ray fluorescence (XRF) maps of two coccolith specimens showing W, Ti, Cr and Mn localized spots. **a, b**) The maps show the distribution of W (in red) with respect to two coccolith specimens Ca (green). W is localized in one or few spots compatible with contamination from the tungsten needle used for coccolith picking. **c, d, e)** Ti, Cr and Mn localized distribution is displayed with respect to specimen Cp4.

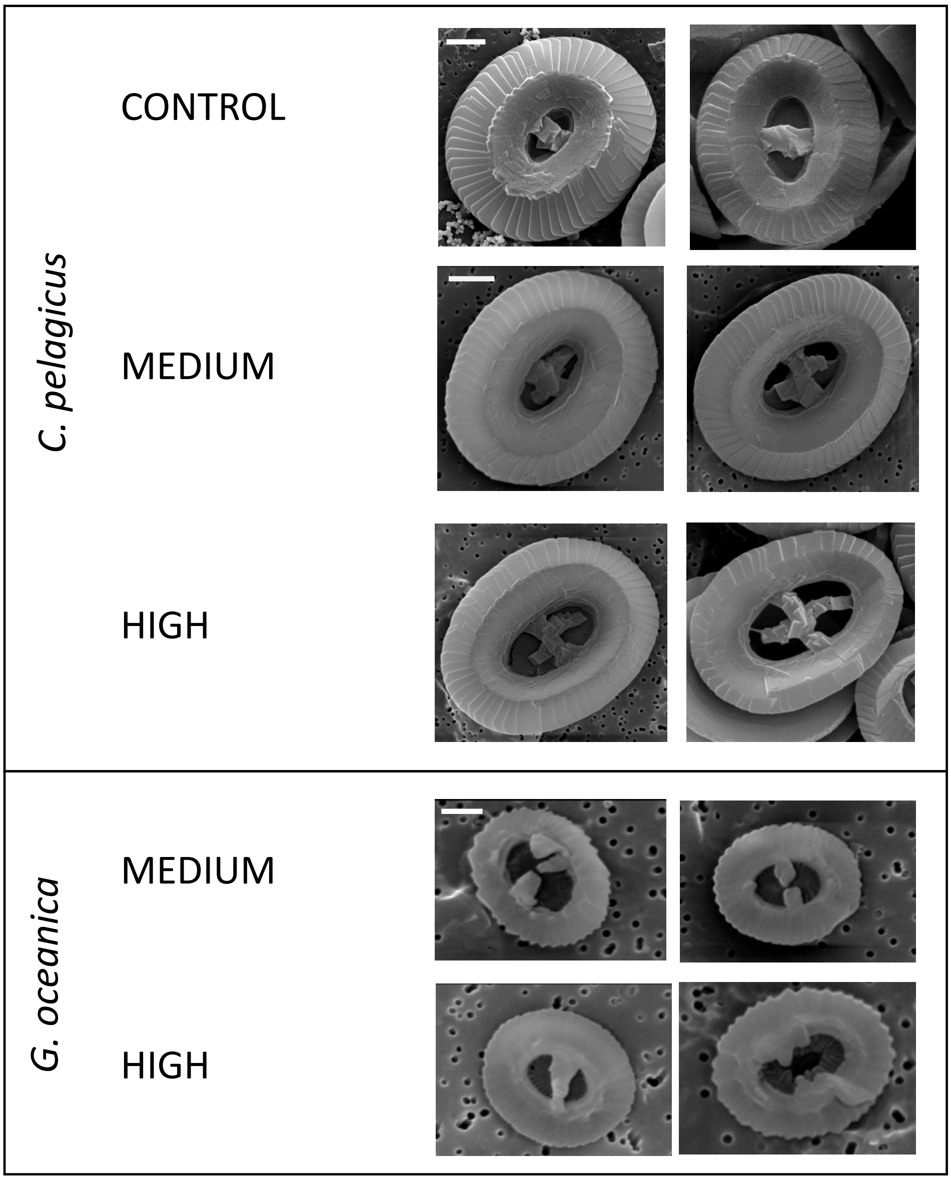

Supplementary Figure_3. Scanning electron microscope (SEM) images of *Coccolithus pelagicus* and *Gephyrocapsa oceanica* specimens taken from the same experiments[2] of the ones studied in this work. For each experiment SEM pictures of two coccolith specimens are presented. SEM pictures were captured from filters. "Control" refers to control conditions, "medium" and "high" refer to specimens cultured under medium and high trace metal (Ni, V, Pb and Zn) concentrations as reported in Faucher et al.[2]. Some of those from M and H experiments show irregularities in the rim and the bridge in *G. oceanica* is missing. The scale bar is 2 μm for all pictures.

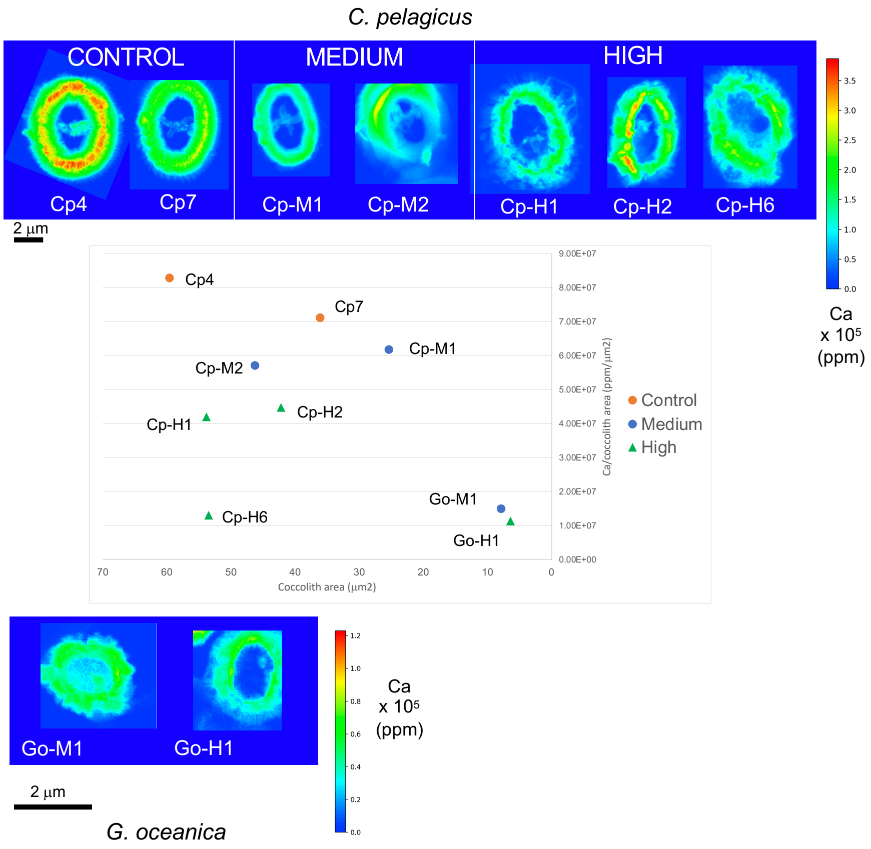

Supplementary Figure_4. ***Coccolitus pelagicus* and *Gephyrocapsa oceanica* Ca /coccolith surface area (ppm/µm²).** The graph reports the average Ca/surface area (ppm/µm²) for each studied *C. pelagicus* and *G. oceanica* specimen. The graph shows that the *C. pelagicus* specimens from the control (C) have higher average Ca/area than the specimens from M and H experiments. *G. oceanica* specimen from M experiment has slightly higher Ca /area than the specimen from H experiment. These data show that *C. pelagicus* coccoliths from M and H experiment are probably thinner compared to coccolith from C. Cp-M2 data must be taken with caution as the coccolith is tilted. Cp = *C. pelagicus*, Go = *G. oceanica*.

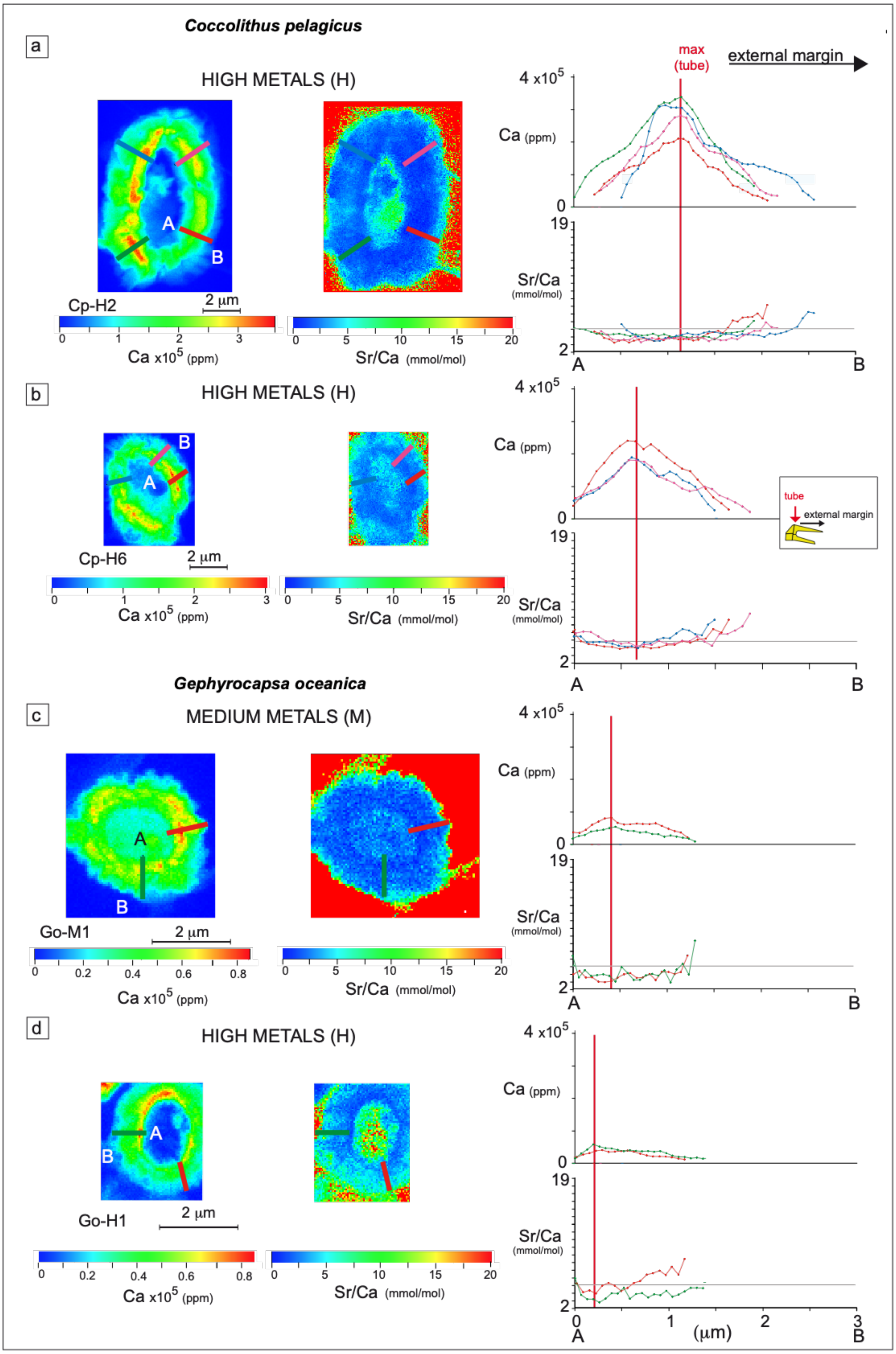
a
Coccolithus pelagicus
HIGH METALS (H)
A
B
Cp-H2
2 μm
Ca x10^5 (ppm)
Sr/Ca (mmol/mol)
max (tube)
external margin
4 x10^5
Ca (ppm)
Sr/Ca (mmol/mol)
b
HIGH METALS (H)
Cp-H6
tube
external margin
Gephyrocapsa oceanica
c
MEDIUM METALS (M)
Go-M1
d
HIGH METALS (H)
Go-H1
(μm)

Supplementary Figure_5. Ca and Sr/Ca profiles across the studied coccolith rim. On the left X-ray fluorescence (XRF) maps are reported for Ca concentrations (ppm) and Sr/Ca ratios in two *Coccolithus pelagicus* and two *Gephyrocapsa oceanica* specimens. The specimens are from **a**) and **b)** high (Cp-H2, Cp-H6, respectively) metals experiments, **c**) medium metal concentration (Go-M1) and **d)** high metal experiment (Go-H1). In each map two to three transects are traced in different colors. Ca x10^5 (ppm) and Sr/Ca (mmol/mol) profiles are reported on the right. The transects were traced only for Ca > 0.2 x10^5 (ppm) thus excluding the background. Considering the Ca concentration to be proportional to the coccolith thickness, the profiles provide indication of the lateral change in coccolith thickness. The Sr/Ca profiles highlight higher mean values in the specimen form H experiment and higher average Sr/Ca values in the external part of the rim of all specimens.

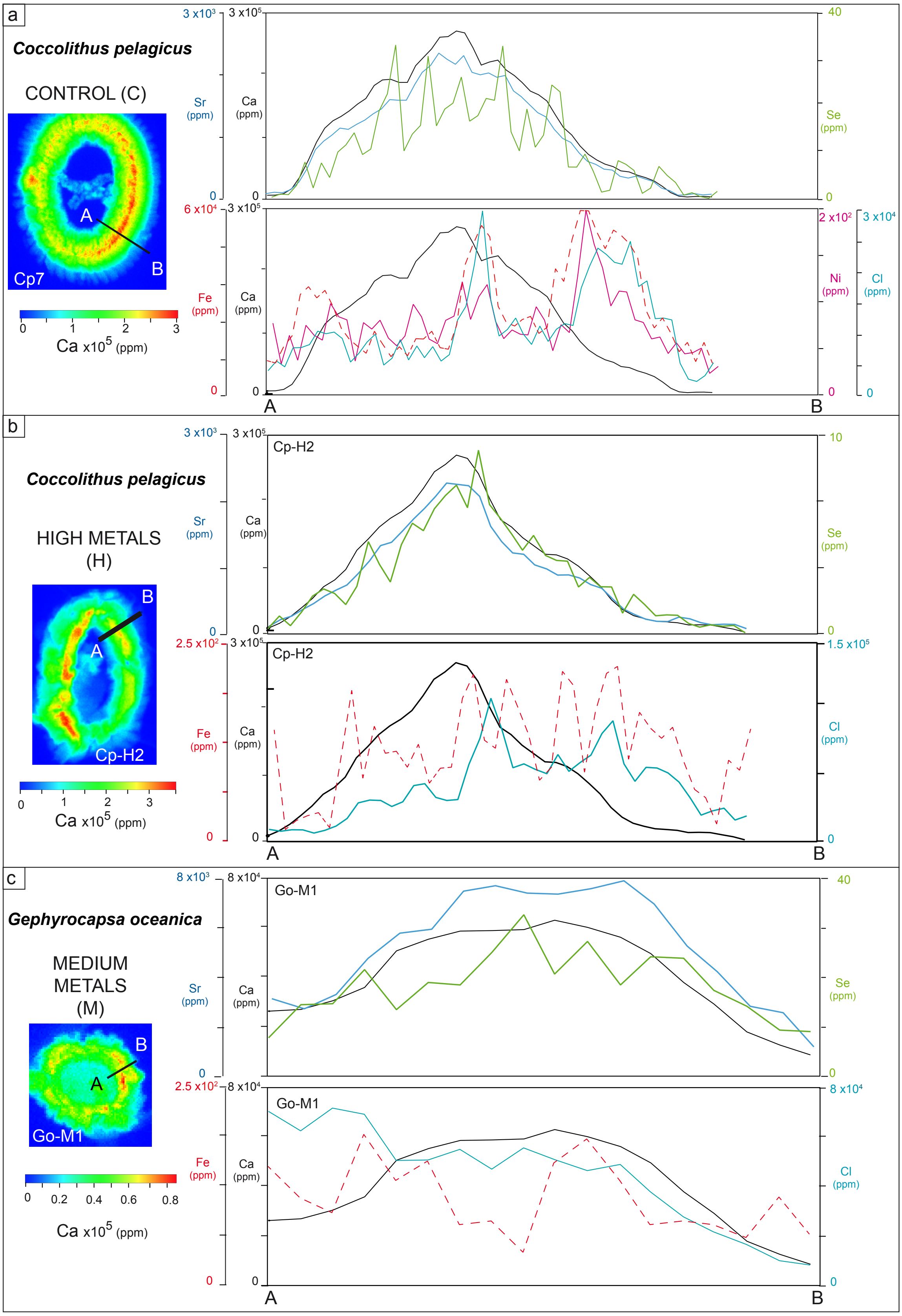

a
Coccolithus pelagicus
CONTROL (C)
A
B
Cp7
Ca x10^5 (ppm)
Sr (ppm)
Ca (ppm)
Se (ppm)
Fe (ppm)
Ni (ppm)
Cl (ppm)
b
Coccolithus pelagicus
HIGH METALS (H)
Cp-H2
c
Gephyrocapsa oceanica
MEDIUM METALS (M)
Go-M1

continues

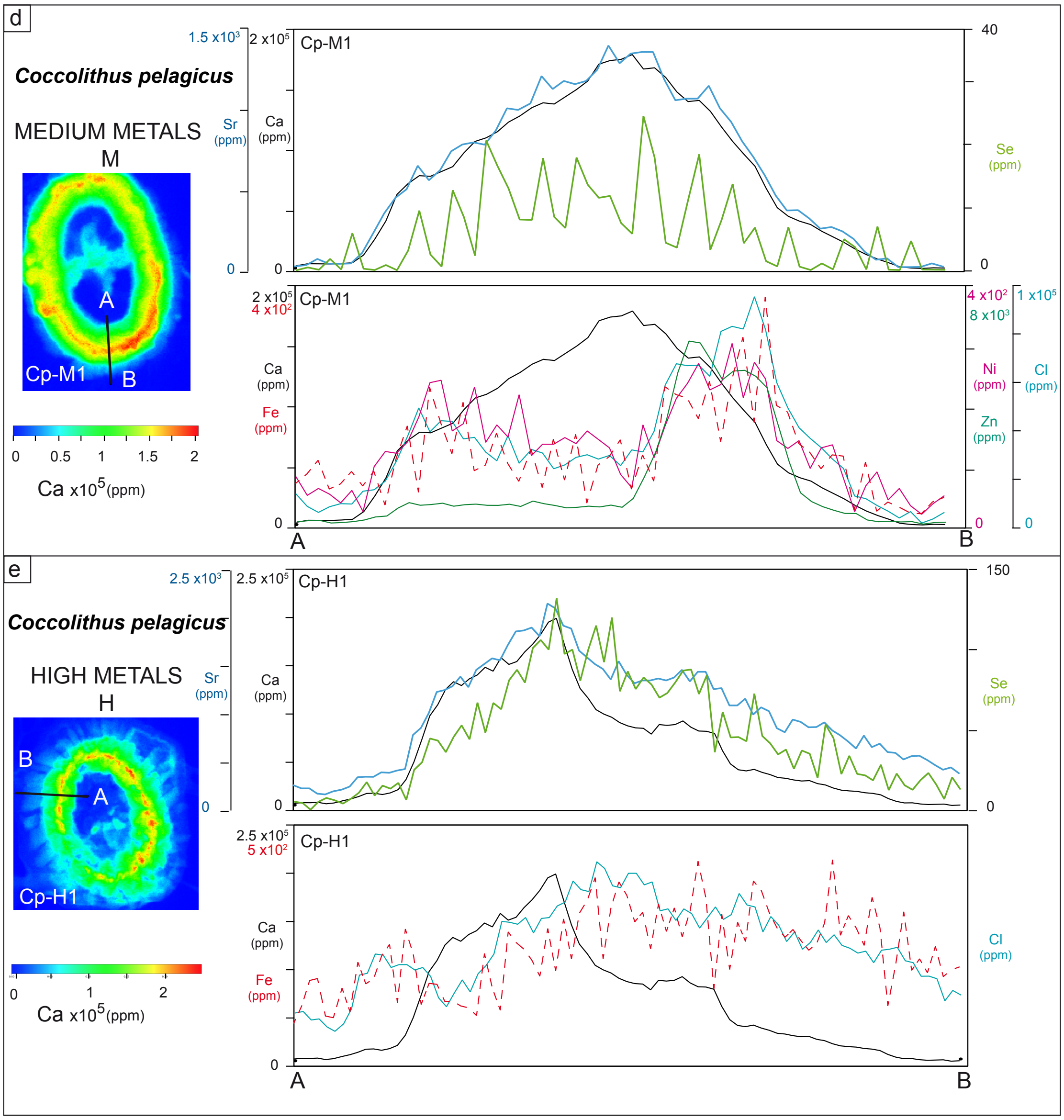

Supplementary Figure_6. Ca, Sr, Se, Ni, Zn, Fe, and Cl profiles across *Coccolithus pelagicus* and *Gephyrocapsa oceanica* coccolith rim. On the left X-ray fluorescence (XRF) maps show the Ca concentrations (ppm) and distribution in **a)** *Coccolithus pelagicus* from the control (Cp4), **b)** *Coccolithus pelagicus* from high metals (Cp-H2) experiment, **c)** *Gephyrocapsa oceanica* from medium metals (Go-M1) experiment, **d)** *Coccolithus pelagicus* from medium metals (Cp-M1) experiment, **e)** *Coccolithus pelagicus* from high metals (Cp-H1) experiment. The transect "A-B" across the coccolith rim is reported in the XRF maps. The upper profiles on the right report Ca (black), Sr (blue) and the elements displaying similar trends such as Se (green). The lower profiles represent Ca (black), Cl (light blue) and the elements with a trend similar to Cl such as Fe (dashed red), Zn (green) and Ni (pink). Se profile is reported in Go-M1 although its concentrations are not fully reliable due to a badly resolved peak spectrum.

Experiment	Specimen	Species	N. of data points	**W/Ca** (mmol/mol)	**Ti/Ca** (mmol/mol)	**Cr/Ca** (mmol/mol)	**Mn/Ca** (mmol/mol)
Control	Cp4	*C. pelagicus*	30026	220.3	374.8	245.4	169.6
	Cp7	*C. pelagicus*	20202	306.6	384.2	351.7	175.1
Medium	Cp-M1	*C. pelagicus*	17574	206.8	362.7	307.0	901.1
	Cp-M2	*C. pelagicus*	35938	657.1	757.1	408.4	516.5
High	Cp-H1	*C. pelagicus*	34158		2106.9	1393.3	
	Cp-H2	*C. pelagicus*	16528		814.9	539.5	
	Cp-H6	*C. pelagicus*	7142			695.9	
Medium	Go-M1	*G. oceanica*	3294		1677.3	1165.7	
High	Go-H1	*G. oceanica*	5538	3470.3	2777.8	1563.9	1457.6

Supplementary Table_1. Element concentrations detected via X-ray fluorescence (XRF) in the studied samples. Average i/Ca (mmol/mol) for each studied sample. Specimens are grouped on the basis of the culture experiment (from Faucher et al.[2]): control conditions, medium and high V, Ni, Zn and Pb concentrations.

Control		Cl	Ca	Fe	Ni	Se
Cp7	Cl					
	Ca	0.26				
	Fe	0.39	0.17			
	Ni	0.18	0.15	0.28		
	Se	0.17	**0.59**	0.13	0.09	
	Sr	0.25	**0.98**	0.17	0.15	**0.59**

Control		Cl	Ca	Fe	Ni	Se
Cp4	Cl					
	Ca	0.29				
	Fe	0.36	0.00			
	Ni	**0.50**	0.30	0.13		
	Se	0.28	**0.65**	0.04	0.27	
	Sr	0.29	**0.98**	0.01	0.30	**0.64**

Medium		Cl	Ca	Fe	Ni	Zn	Se
Cp-M1	Cl						
	Ca	0.10					
	Fe	0.32	0.06				
	Ni	0.34	0.15	**0.56**			
	Zn	**0.52**	0.12	0.24	0.34		
	Se	0.07	**0.44**	0.04	0.10	0.10	
	Sr	0.07	**0.98**	0.04	0.13	0.11	**0.44**

Medium		Cl	Ca	Fe	Ni	Zn	Se
Cp-M2	Cl						
	Ca	0.07					
	Fe	0.06	-0.05				
	Ni	**0.55**	-0.13	0.19			
	Zn	0.29	0.02	0.22	0.18		
	Se	0.13	**0.57**	-0.02	0.02	0.06	
	Sr	0.04	**0.78**	-0.18	-0.10	0.10	**0.46**

Medium		Cl	Ca	Fe	Se
Go-M1	Cl				
	Ca	0.13			
	Fe	0.30	-0.04		
	Se	0.03	0.24	-0.03	
	Sr	0.07	**0.84**	-0.07	0.21

High		Cl	Ca	Fe	Se	Sr
Cp-H1	Cl					
	Ca	0.08				
	Fe	0.28	0.05			
	Se	0.33	**0.73**	0.22		
	Sr	0.22	**0.91**	0.18	**0.80**	

High		Cl	Ca	Fe	Se
Cp-H2	Cl				
	Ca	0.19			
	Fe	0.17	0.13		
	Se	**0.54**	**0.74**	0.17	
	Sr	0.36	**0.97**	0.18	**0.82**

High		Cl	Ca	Fe	Se
Cp-H6	Cl				
	Ca	0.35			
	Fe	0.32	0.12		
	Se	0.00	**0.65**	0.00	
	Sr	**0.43**	**0.96**	0.16	0.29

High		Cl	Ca	Fe	Se
Go-H1	Cl				
	Ca	0.24			
	Fe	0.04	-0.14		
	Se	0.12	**0.37**	-0.07	
	Sr	0.16	**0.37**	0.18	0.15

Supplementary Table_2. Pearson's Correlation Coefficients of the elements detected in the studied coccoliths. Significant coefficients ($p < 0.01$) are in bold.

C. pelagicus							*G. oceanica*	
CONTROL		MEDIUM		HIGH			MEDIUM	HIGH
Cp7	Cp4	Cp-M1	Cp-M2	Cp-H1	Cp-H2	Cp-H6		
-0.133	-0.411	-0.109	-0.514	-0.681	-0.561	-0.624	-0.708	-0.666

Supplementary Table_3. Pearson's Correlation Coefficients calculated for Sr/Ca ratios vs Ca concentrations in the studied samples. The dataset shows that there is no correlation between Sr/Ca and Ca although in M and H experiments r is slightly higher.

	Control	Low	Medium<br>$\mu mol\ L^{-1}$	High	Extreme
$FeCl_3 \cdot 6H_2O$	11.7	11.7	11.7	11.7	11.7
$Na_2 \cdot 2H_2O$	11.7	11.7	11.7	11.7	11.7
$CuSO_4 \cdot 5H_2O$	0.04	0.04	0.04	0.04	0.04
$Na_2MoO4 \cdot 2H_2O$	0.03	0.03	0.03	0.03	0.03
$CoCl_2 \cdot 6H_2O$	0.04	0.04	0.04	0.04	0.04
$ZnSO_4 \cdot 7H_2O$	0.08	0.16	0.16	0.96	8.08
Pb	–	0.01	0.08	0.8	
$NiCl_2 \cdot 6H_2O$	–	0.08	0.08	0.8	8.00
$VOSO_4$	–	0.08	0.08	0.8	8.00
EDTA	11.7	11.7	11.7	11.7	11.7

Supplementary Table_4. Trace metal concentrations in the growth medium of the different treatments from Faucher et al. [2]. In this work the studied specimens come from the control, medium and high experiments. The artificial seawater medium was added with 10 nmol kg^{-1} of SeO_2 (Faucher et al. [2]).

Supplementary references

1. Solé, V.A., Papillon, E., Cotte, M., Walter, Ph., Susini, J. A multiplatform code for the analysis of energy-dispersive X-ray fluorescence spectra. *Spectrochim. Acta* Part B 62 63-68 (2007).
2. Faucher, G., Hoffmann, L., Bach, L. T., Bottini, C., Erba, E. & Riebesell, U. Impact of trace metal concentrations on coccolithophore growth and morphology: laboratory simulations of Cretaceous stress. *Biogeosciences* 14, 3603-3613 (2017).
